# The adsorptive behaviour of kaolinite to sodium dodecyl benzene sulphonate and the structural variation of kaolinite

**DOI:** 10.1038/s41598-021-81283-8

**Published:** 2021-01-19

**Authors:** Xiaoming Ni, Zheng Zhao, Zhiheng Li, Quanzhong Li

**Affiliations:** 1 School of Energy Science and Engineering, Henan Polytechnic University, Jiaozuo, 454000 China; 2 Mining Engineering Department, Shanxi Institute of Technology, Yangquan, 045000 China; 3 China Coal Technology Engineering Group Chongqing Research Institute, Chongqing, 400037 China

**Keywords:** Mineralogy, Coal, Natural gas

## Abstract

Analysis of the adsorptive behaviour of kaolinite to sodium dodecyl benzene sulphonate (SDBS) at different concentrations can provides a basis for selecting the best concentration. The adsorptive capacity and adsorptive behaviour of kaolinite to SDBS at different concentrations were studied using ultraviolet spectrophotometer, pseudo-first-order adsorption kinetics model, and pseudo-second-order adsorption kinetics model. Scanning electron microscopy with energy dispersive spectrometry (SEM–EDS), X-ray diffraction (XRD), and infrared spectroscopy (FTIR) were used to study the variation characteristics of surface structure, crystallinity indices, and main functional groups on kaolinite before, and after, adsorption. The results show that as the SDBS concentration increase, the adsorptive capacity of kaolinite to SDBS increase. The adsorption process can be accurately fitted by the pseudo-secondary adsorption kinetic model, which means the adsorptive behaviour was mainly chemical in origin. The adsorption of SDBS by kaolinite mainly occurs on the surface. The solidification, lamellar aggregation, and crystallinity index of kaolinite are more obvious after the adsorption of SDBS, but the interlayer spacing of kaolinite did not change to any significant. After the adsorption of SDBS, the intensity ratio of 1000–1008 cm^−1^ bands changed significantly, indicating the change of the chemical environment, and the adsorptive behaviour was chemical.

## Introduction

Coal rocks, sandstone and shale reservoirs often contain clay minerals such as kaolinite. When hydraulic fracturing is carried out on these reservoirs, clay minerals and fracturing fluids in the reservoirs are prone to produce effects such as velocity sensitivity, alkali sensitivity and salt sensitivity, which have a great impact on the effect of reservoir reconstruction. After adding surfactant to the fracturing fluid, surface active agents are often adsorbed on clay minerals^1–4^ to change the surface structure of clay minerals and reduce the above effects. Sodium dodecyl benzene sulphonate (SDBS) is a commonly used anionic surfactant^5^, discovering the adsorption characteristics of SDBS on the surface of kaolinite minerals can provide a basis for the selection of the optimum concentration thereof.

The adsorptive behaviour of solids to surfactants can be described by fitting the adsorption process at different times using classical models. Among them, the methods for measuring the adsorptive capacity are mainly spectrophotometric and liquid chromatographic techniques. The spectrophotometric measurement method involves supernatant being collected from centrifuged liquid for measurement of absorbance. Then the adsorptive capacity is obtained according to the relationships between concentration and absorbance. This method, due to its ease of operation, is a common method used for testing the adsorptive capacity^6,7^. Liquid chromatography is based on the small differences in physical and chemical properties with regard to the solubility, adsorptive capacity, and ion exchange capacity of substances. These should be combined with the differences between the partition coefficient of mobile and fixed phases: the various substances to be measured were separated through the distribution of their fixed and mobile phases, according to the difference in relative solubility between both phases^8,9^. The adsorptive capacity (for quantitative evaluation) also requires spectrophotometry. The commonly adsorption kinetic models used in fitting adsorption processes include the pseudo-first-order adsorption kinetic model, pseudo-second-order adsorption kinetic model, Elovich kinetic model, and Bangham adsorption rate model^10–15^. It is generally believed that pseudo-first-order adsorption kinetics model is more accurate for the adsorption process fitting of physical adsorption, while the pseudo-second-order adsorption kinetics model is more accurate when simulating chemical adsorption^16–18^. The structural change characteristics of minerals after adsorption of anionic surfactant were mainly detected by scanning electron microscopy with energy dispersive spectrometry (SEM–EDS), X-ray diffraction (XRD), and infrared spectroscopy (FTIR)^19–24^.

Herein, the adsorptive capacity of kaolinite to SDBS at different concentrations under different time was tested with the help of ultraviolet spectrophotometry, and the adsorption type of SDBS was determined by fitting the adsorption process with the classical model. SEM–EDS, XRD, and FTIR were used to test the differences in element content, layer spacing, and main functional groups before and after adsorption. The structural changes of kaolinite were thus obtained. The research can provide a basis for selecting the best concentration of SDBS.

## Experimental samples

The main samples required for the experiment were the anionic surfactant SDBS and kaolinite minerals. The SDBS (analytically pure) used in the experiment was produced by Fangzheng Reagent Factory, Beichen District, Tianjin, China. In the SDBS (C_18_H_29_NaO_3_S), the active substance content is ≥ 90%, the sodium salt content is ≤ 7%, and the moisture content is ≤ 3%. The block sample had good crystallinity and few impurities (the kaolinite content is greater than 95%). The kaolinite sample was pulverised and then tested by X-ray diffraction and energy spectral analysis. The chemical formula of the kaolinite powder was Al_2_Si_2_O_5_(OH)_4_. The oxidic composition and mass fraction of each mineral in kaolinite are shown in Table 1.

**Table 1:** The oxidic composition of kaolinite.

Mineral	Component	Al_2_O_3_	SiO_2_	Fe_2_O_3_	TiO_2_	MgO	CaO	Na_2_O	K_2_O	Others
Kaolinite	Mass fraction (%)	39.2	43.67	0.32	1.98	0.068	0.01	0.028	0.094	14.63

## Research methods

### The adsorptive behaviour of kaolinite to SDBS

1. The test for adsorptive capacity.
  1. Sample preparation
    We weighed 2.000 g of kaolinite (three pieces) on a precision balance and placed them into a conical bottle. 200 mL of SDBS solutions with concentrations of 3 mmol/L, 4 mmol/L, and 5 mmol/L were thus prepared.
  2. Absorbance test
    The three different concentrations of SDBS solutions were poured into the conical flask containing kaolinite and then stirred on a magnetic agitator (set the operating temperature as 25 °C during the experiment). The upper clear solution was filtered after stirring for 0 min, 20 min, 40 min, 60 min, 90 min, and 120 min, respectively, then the absorbance of the filtrate was measured by 201 UV spectrophotometer (The test wavelength of the ultraviolet spectrophotometer ranged from 190 to 1100 nm and the wavelength accuracy was ± 0.8 nm).
  3. Adsorptive capacity calculation
    The absorbance peak of SDBS occurs at about 223 nm, and when the solution concentration is 3–5 mmol/L, the absorbance peak has a good linear relationship with the concentration of SDBS^5^:
    1 $$C = 4.80071A + 0.4465$$
    where, *C* is concentration of SDBS, mmol/L; *A* represents the peak wavelength of absorbance.
    According to the absorbance test results of filtered filtrate, in combination with formula (1), the adsorptive capacity can be calculated as follows:
    2 $${q_t} = \frac{{({C_0} - {C_t})V}}{m}$$
    where, *q*_t_ is the adsorptive capacity at time *t*, mmol/g; *C*_0_ represents the initial concentration, mmol/L; *C*_t_ is the concentration at time *t*, mmol/L; *V* is the volume of the solution, L; *m* is the mass of the kaolinite, g.
2. Determination of the adsorptive behaviour of kaolinite to SDBS
  The adsorptive behaviour of kaolinite to SDBS was investigated mainly by comparing the adsorption process with the classical adsorption kinetic model.
  Wherein, the fitting formula of pseudo-first-order adsorption dynamics equation to characterise physical adsorption is as follows^5^:
  3 $${q_t} = {q_e}\left[ {1 - \exp ( - {k_1}t)} \right]$$
  where, *q*_e_ is the equilibrium absorption capacity, mmol/g; *k*_1_ is the first-order adsorption constant, min^−1^.
  The fitting formula of pseudo-second-order kinetic model to characterise chemical adsorption is as follows^5^:
  4 $${q_{\text{t}}} = {k_2}{q_e}^2t/\left( {1 - {k_2}{q_e}t} \right)$$
  where, *k*_2_ is the second-order adsorption constant, g·mmol^−1^·min^−1^; *k*_2_*q*_e_^2^ is the initial adsorption time, mmol·g^−1^·min^−1^.

### The structural variation of kaolinite before and after adsorption

Centrifuge the mixture of kaolinite and different concentrations SDBS solutions after adsorption, and dry the precipitate in a thermostatic drying chamber. Together with the original kaolinite, respectively, marked as the Original sample, 3 mmol/L SDBS after adsorption, 4 mmol/L SDBS after adsorption, 5 mmol/L SDBS after adsorption, and in preparation for the experiment.

1. SEM–EDS testing before and after adsorption
  The surface morphology of kaolinite before and after adsorption of SDBS were studied by using the SEM–EDS, which was from Carl Zeiss AG, Germany. The experiment was carried out in Henan Polytechnic University. The acceleration voltage of the experiment was 0.2–30 kV, and the apparatus able to analyse elements from Be to U. The specific methods are as follows:
  1. Gold plating for the four kaolinite samples before and after adsorption.
  2. Observe the four gold-plated samples under SEM with different magnifications, and assay the element content by EDS.
  3. Analyse the changes of surface morphology and element content before and after adsorption.
2. XRD testing before and after adsorption
  XRD was used to test the variations of surface structure and crystallinity index of kaolinite before and after adsorption of SDBS. The experiment was carried out in Henan Polytechnic University using D8 X-ray diffractometer manufactured by BRUKER-AXS, Germany. The light source adopts X-ray phototube copper target radiation (Cu, λ 1.5406A). The divergent slit, anti-scatter slit, Sola slit, and receiving slit were 1.0 mm, 1.0 mm, 2°, and 0.2 mm, respectively. The Angle measurement range of this experiment is 2° ~ 90° and the scanning mode was step-scan with a step length and scanning speed of 0.1° and 3 s/step. The specific testing process is as follows:
  1. Grind the four samples to less than 200 mesh and place them into the test tray for compaction.
  2. Test the four samples by XRD, and compare the difference before and after adsorption.
  3. Calculate the layer spacing
    According to the Bragg Equation^25^ and the 001 peak position *θ*_001_, the layer spacing *d*_001_ can be can be calculated by the following formula:
    5 $${d_{001}} = \frac{\lambda }{{2\sin {\theta_{001}}}}$$
  4. Calculate the crystallinity indices
    The crystallinity indices *H*_i_ can be calculate from XRD data^26^:
    6 $${H_i} = \frac{A + B}{{A_t}}$$
    where, A and B are the heights of two adjacent diffraction reflections, and At is the distance between the highest reflection of the two adjacent diffraction reflections and the back bottom line.
3. FTIR testing before and after adsorption
  FTIR was used to test the functional group changes of kaolinite before and after adsorption of SDBS. The experiment was carried out in Henan Polytechnic University using 70 FTIR manufactured by BRUKER VERTEX, Germany. The measurement ranges of spectral area, step-scan time resolution, and resolution were 4000 to 400 cm^−1^, 5 ns, and 0.4 cm^−1^, respectively. The specific method is as follows:
  1. Weigh 0.010 g of the four kaolinite samples, and mix them with 1.500 g KBr in a ratio of 1:150, respectively.
  2. Grind them using an agate mortar until the sample particles were less than 200 mesh equivalent spherical diameter, then place into the thermostatic drying chamber at 80 °C for 24 h.
  3. Grind the dry mixed powder in an agate mortar for 1–2 min, and form tablets for infrared spectroscopy.

## Experimental results

### Analyses on the adsorptive behaviour of kaolinite to SDBS

**Figure 1:**
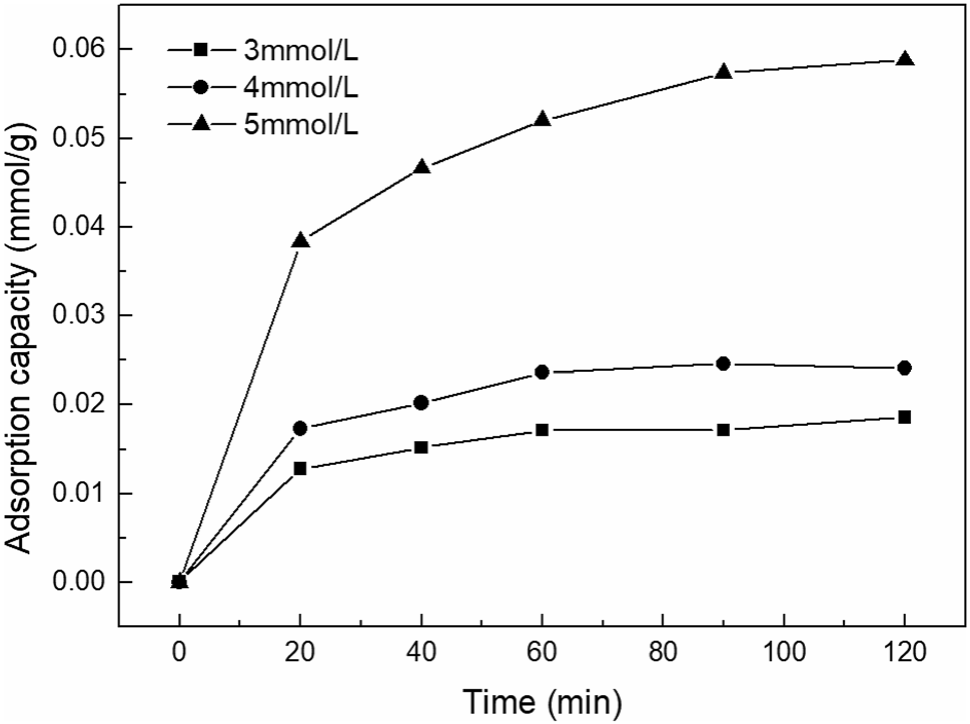
Adsorptive capacity of SDBS on kaolinite at various SDBS concentration under different adsorption time.

The adsorptive capacity of kaolinite to SDBS was calculated according to the experimental design, and the results are shown in Fig. 1.

In Fig. 1, the adsorptive capacity increased with concentration, and the adsorptive capacity of kaolinite to SDBS with three different concentrations increased slowly over time.

According to formulae (3) and (4), different models were used to fit the experimental process. The fitting results and main adsorption parameters are listed in Table 2.

**Table 2:** Fitting results of pseudo-first order and pseudo-second order model at different concentrations.

Concentration of SDBS	The pseudo-first order model	*q*_e_(exp)/mmol/g	*q*_e_(cal)/mmol/g	*k*_1_/min^−1^
3 mmol/L	*q*_t_ = 0.01772 × (1−e^−0.05844×t^)	0.0186	0.0177	0.058
4 mmol/L	*q*_t_ = 0.02416 × (1−e^−0.05714×t^)	0.0246	0.0241	0.057
5 mmol/L	*q*_t_ = 0.05722 × (1−e^−0.04906×t^)	0.0588	0.0572	0.049

Concentration of SDBS	The pseudo-second order model	*q*_e_(exp)/mmol/g	*q*_e_(cal)/mmol/g	*k*_2_/ mmol·g^−1^·min^−1^
3 mmol/L	*q*_t_ = t/(582.6025 + 50 × t)	0.0186	0.0182	4.291
4 mmol/L	*q*_t_ = t/(430.3774 + 36.75119 × t)	0.0246	0.0241	3.138
5 mmol/L	*q*_t_ = t/(231.368 + 15.11944 × t)	0.0588	0.0587	0.988

A variety of error analysis method, including the root mean square error (RMSE), chi-squared distribution (*χ*^2^), G party inspection (*G*^2^), error sum of squares (ERRSQ), composite relative error function (HYBRZD), Marquardt proportion standard deviation derivative (MPSD), average relative deviation (ARE), sum of absolute error (EABS), absolute pose error (APE), Akaike information criterion (AIC), Mallows *C*_p_ (Mallows), etc., were used in the discussion of the correlation of fitting (Table 3).

**Table 3:** Error analysis of the fitting results of kaolinite adsorption kinetics under different SDBS concentrations.

Error Function Model	Pseudo-first order kinetic model			Pseudo-second order adsorption model		
	3 mmol/L	4 mmol/L	5 mmol/L	3 mmol/L	4 mmol/L	5 mmol/L
RMSE(10^–3^)	0.71	0.91	2.34	0.42	0.74	0.93
χ^2^(10^–4^)	1.25	1.62	4.76	0.42	0.96	0.73
G^2^(10^–4^)	3.00	4.50	15.44	0.81	0.69	2.41
ERRSQ(10^–6^)	2.01	3.32	21.82	0.72	2.18	3.44
HYBRD(10^–4^)	1.25	1.67	4.72	0.42	0.97	0.73
MPSD(10^–3^)	7.88	8.45	10.44	2.49	4.33	1.60
ARE	0.17	0.16	0.21	0.11	0.13	0.07
EABS (10^–3^)	2.80	3.18	9.95	1.75	3.02	3.80
APE%	3.52	3.14	4.18	2.10	2.69	1.59
AIC_C_	− 81.46	− 78.46	− 67.15	− 87.65	− 80.97	− 78.25
Mallows(10^–3^)	2.83	3.63	9.34	1.69	2.95	3.70

From the calculated results of various error functions in Tables 1 and 2, the pseudo-secondary adsorption kinetic model returned a smaller error and was more consistent with the adsorption performance, indicating that the adsorption on the kaolinite surface of SDBS is mainly chemical. The adsorption constant decreases gradually with the increased concentration of SDBS, this is mainly because the repulsion between adsorbed molecules and free SDBS molecules in solution increases with the increase of SDBS concentration.

### The structural variation of kaolinite before, and after, adsorption

#### Surface morphology variation characteristics of kaolinite before, and after, adsorption

**Figure 2:**
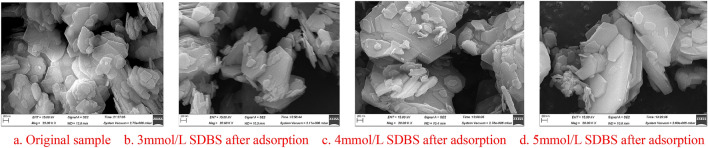
SEM test chart before and after the adsorption of kaolinite, (**a**) Original sample (**b**) 3 mmol/L SDBS after adsorption (**c**) 4 mmol/L SDBS after adsorption (**d**) 5 mmol/L SDBS after adsorption.

**Figure 3:**
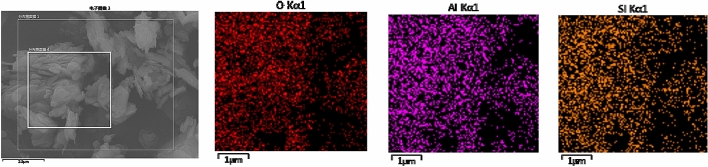
EDS scan of samples before adsorption of kaolinite.

**Figure 4:**
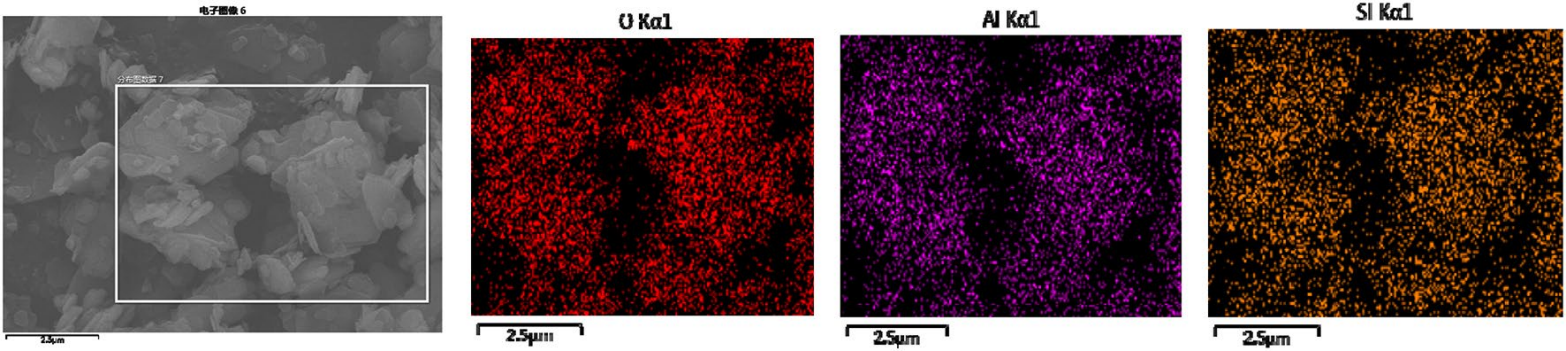
EDS scan of kaolinite after the adsorption of 4 mmol/L SDBS.

**Figure 5:**
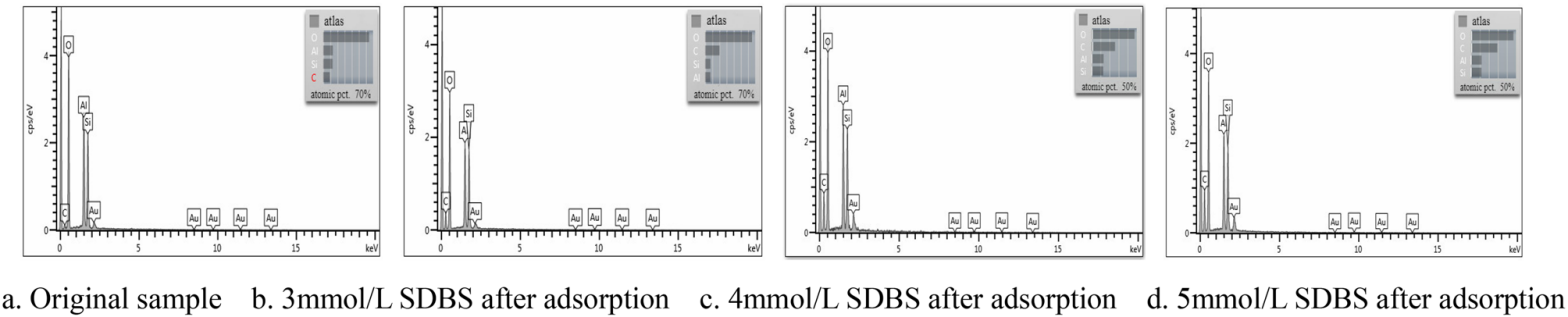
EDS scan of kaolinite before and after adsorption. (**a**) Original sample (**b**) 3 mmol/L SDBS after adsorption (**c**) 4 mmol/L SDBS after adsorption (**d**) 5 mmol/L SDBS after adsorption.

SEM scanning results for part of the samples before and after the adsorption of SDBS by kaolinite are shown in Fig. 2, and the EDS test results are shown in Figs. 3, 4, 5.

Figure 2 shows that the structure of kaolinite layers after adsorption of SDBS is clearer, and the phenomenon of plate formation is more obvious. As can be seen from Figs. 3, 4, 5: O, Al, and Si are the main elements found on the surface of the kaolinite, while a small amount of C mainly originates from the SEM sample holder or some carbonate impurities thereon. According to the results of energy spectral analysis, the content of element O is higher, Al and Si have little difference in their contents. It is believed that the unit structure of kaolinite is composed of a layer of silicon-oxygen tetrahedron and a layer of aluminium-oxygen octahedron, with a high crystallinity therein^27,28^, which meets the experimental requirements.

#### Characteristics of interlayer change before, and after, kaolinite adsorption

**Figure 6:**
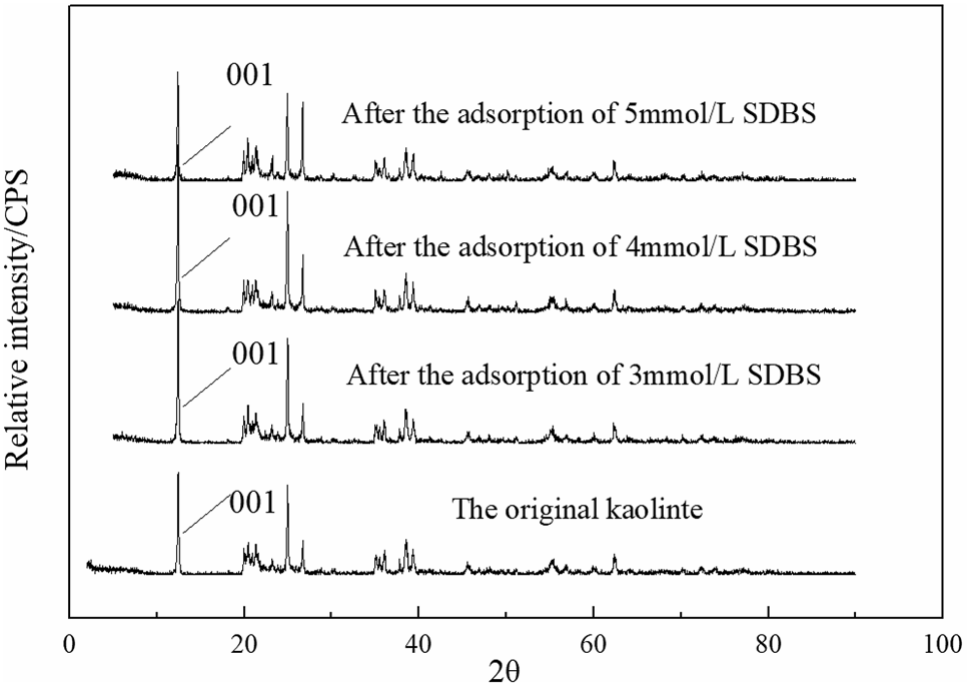
Comparison of XRD test results.

The interlayer change before and after kaolinite adsorption is shown in Fig. 6.

According to the XRD test results of kaolinite under the original conditions and after absorbing SDBS of different concentrations (Fig. 6), it can be found that no new characteristic reflections were generated for kaolinite before and after adsorption. The 2*θ* of 001 reflection for kaolinite samples in the original state and after absorbing 3 mmol/L, 4 mmol/L, and 5 mmol/L SDBS are basically consistent, indicating that the kaolinite layer spacing D001 crystallinity indices did not change significantly. The SDBS was hardly adsorbed between layers of kaolinite, and the adsorption mainly occurred on the surface.

**Figure 7:**
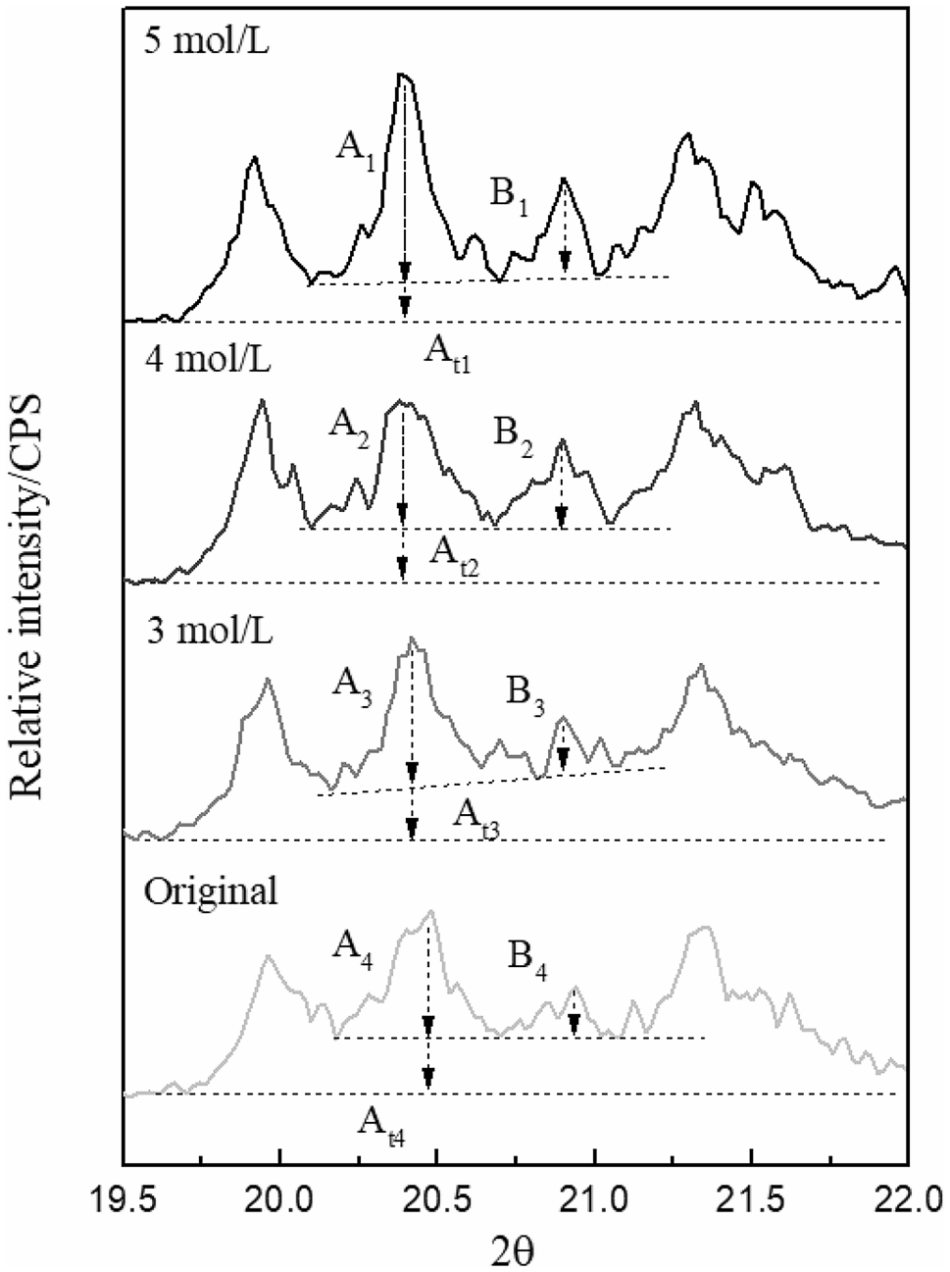
Calculation of crystallinity indices of kaolinite.

According to Formula (6), the crystallinity indices of kaolinite under the original state and after absorbing different concentrations of SDBS is calculated (Fig. 7). The required parameters and calculation results are shown in Table 4. The results shows that after the adsorption of SDBS, the crystallinity indices of kaolinite increased significantly, and when the SDBS concentration was 4 mmol/L, the crystallinity indices reached the maximum, which is consistent with the SEM observation results.

**Table 4:** The required parameters and calculation results of crystallinity indices.

Sample	A	B	A_t_	H_i_
Original sample	51.0	21.0	74.0	1.06
3 mmol/L SDBS after adsorption	67.5	27.0	87.0	1.09
4 mmol/L SDBS after adsorption	54.5	42.5	73.0	1.33
5 mmol/L SDBS after adsorption	83.0	41.5	99.0	1.26

#### Changes in major functional groups before, and after, kaolinite adsorption

**Figure 8:**
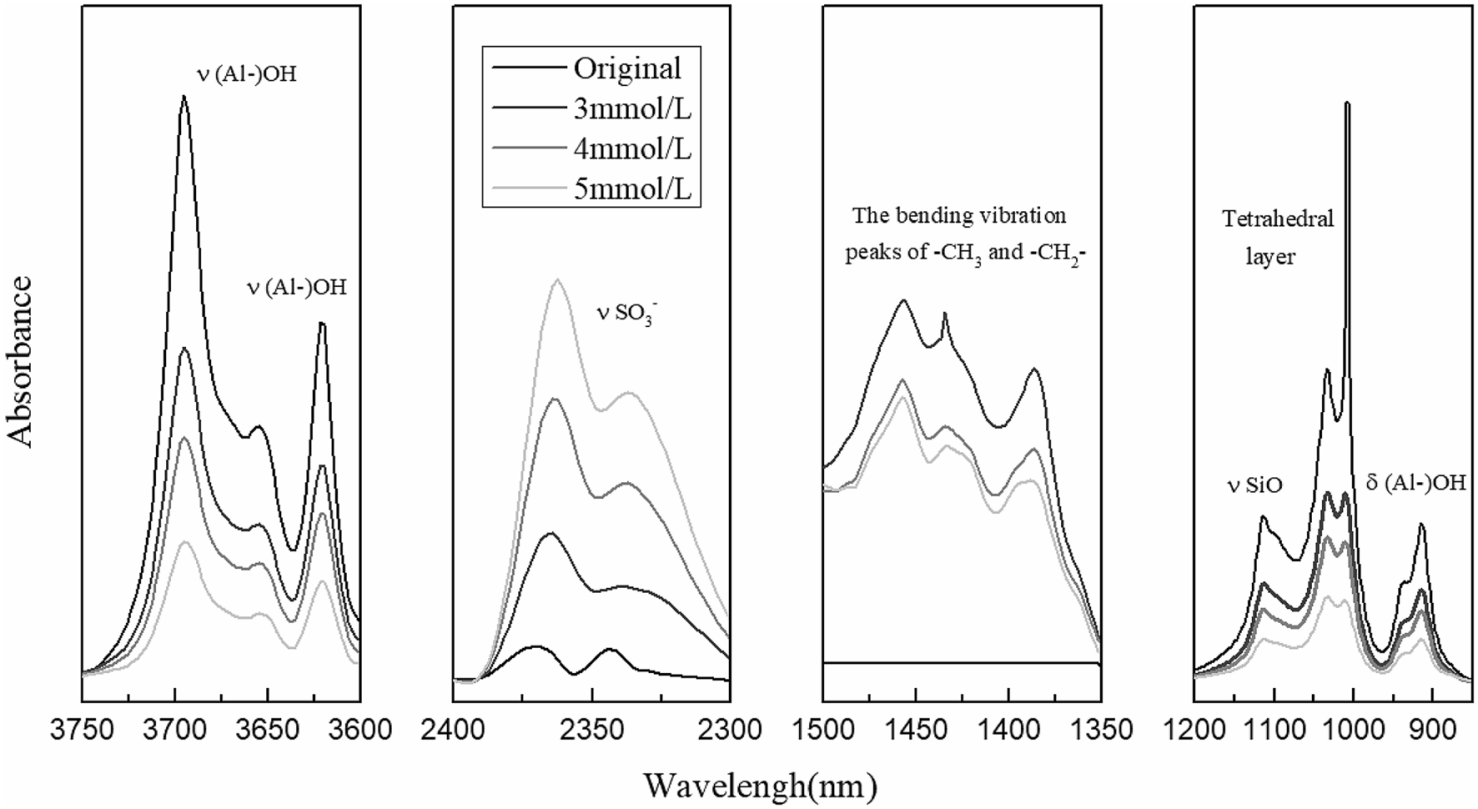
Infrared spectrum testing results under different concentration of concentration solution.

According to the experimental method (“Research methods”), the infrared spectrum testing results of kaolinite before and after adsorption were obtained (Fig. 8).

The infrared spectrum test results of kaolinite under the original conditions and after absorbing SDBS of different concentrations were compared (Fig. 8). It is found that after absorbing SDBS on kaolinite, the bending vibration peaks of –CH_3_ and –CH_2_– appeared at 1475–1350 cm^−1^ indicating that kaolinite has adsorbed SDBS. The intensity ratio of νSO_3_^-^ at 2370 cm^−1^ increased significantly, which also proved the existence of adsorption phenomenon. The intensity ratio of the internal hydroxyl at 3620 cm^−1^ and the external hydroxyl at 3690 cm^−1^ was significantly weakened, and with the increase of SDBS concentration, the weakening was more obvious, which indicates that chemical combination occurred between SDBS and the hydroxyl of the kaolinite. The significant decrease of νSiO at 1107 cm^−1^ and that of δ(Al–)OH at 914 cm^−1^ suggested that chemical adsorption occurred between SDBS and Si and Al. The intensity ratio of 1000–1008 cm^−1^ bands of the tetrahedral layer changed significantly, indicating the change of the chemical environment after kaolinite adsorbed SDBS.

## Conclusion

1. The linear relationship between absorbance and concentration-absorbance measured by spectrophotometer was used to calculate the adsorptive capacity and adsorptive behaviour of SDBS on the surface of kaolinite at different concentrations (3 mmol/L, 4 mmol/L, and 5 mmol/L) at different times. The results showed that the higher the SDBS concentration, the higher the adsorptive capacity, but the higher the concentration, the lower the initial adsorption rate. Its adsorption is best described by the pseudo-secondary adsorption kinetics model, suggesting mainly chemical adsorption.
2. The results of SEM–EDS, XRD, and FTIR show that SDBS was mainly adsorbed on the surface of kaolinite. After the adsorption of SDBS, the solidification and lamellar aggregation of kaolinite were more obvious, the crystallinity index of kaolinite increased significantly, and the crystallinity index reached the maximum when the SDBS concentration was 4 mmol/L. In addition, the intensity ratio of 1000–1008 cm^−1^ bands of the tetrahedral layer changed significantly, indicating that the adsorptive behaviour was chemical.
3. The adsorption behaviours of kaolinite mineral to SDBS with different concentrations were identified, however, rock contains kaolinite, montmorillonite, illite, and other clay minerals; mineral concentration, formation temperature, formation water pH, and composition are likely to affect the adsorption behaviour of SDBS. Future work should focus on analysis of the clay under different environments and the adsorption mechanisms of dodecyl benzene sulphonic acid sodium.

## Data Availability

The data used to support the findings of this study are included within the article.
